# Ingestion of Exopolymers from* Aureobasidium pullulans* Reduces the Duration of Cold and Flu Symptoms: A Randomized, Placebo-Controlled Intervention Study

**DOI:** 10.1155/2018/9024295

**Published:** 2018-05-30

**Authors:** Jong-Min Lim, Eunju Do, Dong-Chan Park, Go-Woon Jung, Hyung-Rae Cho, Seo-Young Lee, Jae Wook Shin, Kyung Min Baek, Jae-Suk Choi

**Affiliations:** ^1^Glucan Corp., #305 Marine Bio-Industry Development Center, 7 Hoenggye-gil, Ilgwang-myeon, Busan 46048, Republic of Korea; ^2^Korean Medicine Industry Support Center, Daegu Technopark, 136 Sincheondong-ro, Suseong-gu, Daegu 42158, Republic of Korea; ^3^Department of Korean Medicine for Internal Medicine, Daegu Korean Medicine Hospital of Daegu Haany University, 136 Sincheondong-ro, Suseong-gu, Daegu 42158, Republic of Korea; ^4^Major in Food Biotechnology, Division of Bioindustry, College of Medical and Life Sciences, Silla University, 140, Baegyang-daero 700beon-gil, Sasang-gu, Busan 46958, Republic of Korea

## Abstract

**Aim:**

The objective of the study was to assess the efficacy of exopolymers from* Aureobasidium pullulans *(EAP) on the incidence of colds and flu in healthy adults.

**Methods:**

We conducted a randomized, double-blind, placebo-controlled study at the onset of the influenza season. A total of 76 subjects (30–70 years of age) were recruited from the general population. The subjects were instructed to take one capsule per day of either EAP or a placebo for a period of 8 weeks. The duration of cold and flu symptoms, a primary variable in assessing effectiveness, and serum cytokine levels as well as WBC counts as secondary variables were also evaluated.

**Results:**

EAP was associated with a statistically significant decrease in the duration of cold and flu symptoms, a primary variable in assessing effectiveness. Although cold and flu symptom levels were not significantly different at a significance level of 5%, the cold and flu symptom levels of the EAP group were less severe compared to the placebo group. No statistically significant changes of serum cytokine levels as well as WBC counts were observed.

**Conclusion:**

The results showed that EAP is a useful pharmaceutical and functional food material for preventing and treating colds and flu.

## 1. Introduction

The immune system has evolved to protect multicellular organisms, including humans, from pathogens. Immunostimulatory effects are regulatory actions that counteract reduced immune responses caused by immunodeficiency diseases, viral infections, malnutrition, tumors, and aging. Two strategies have been developed to stimulate the immune system. One method is to increase antigen-specific or nonspecific immune responses, and the other method involves the use of appropriate immunosupplements for antigen administration [1].

Respiratory viruses are among the most infectious pathogens in humans, and many differences occur in the kinds of pathogens, their antigenicities, and their worldwide infection patterns. Recently, respiratory diseases such as Middle East respiratory syndrome, severe acute respiratory syndrome, and the flu caused by novel swine-origin influenza A strains have emerged and begun to spread rapidly [2–4]. As the associated respiratory viruses show very strong tendencies to spread, there is a growing interest in foods that promote immune functions as well as a global monitoring system for influenza [5].

The results of several researches have revealed a positive correlation between food nutrition and the control of human immune functions, and various foods have been recognized as functional foods that can improve immune functions. These food materials may be derived from vegetables [6], seafood [7], mushrooms [8], microbes such as lactic acid bacteria [9], chitosan [10], and peptides [11].

*β*-1,3/1,6-glucan is derived from yeast cell walls and modulates many* in vivo *and* in vitro *activities [12, 13]. Its main immunopharmacological activities [14–16] are associated with antitumor effects [17], increased host resistance to viral, bacterial, and parasitic infections [18], and adjuvant effects [19]. Proximate composition of extracellular polysaccharides of* Aureobasidium pullulans* is carbohydrate 79.5%, sugar 3.7%, crude protein 4.2%, and moisture 3.2%. Namely, above 80% of the proximate composition is polysaccharides and there is a small amount of protein and fat (personal comm. Dr. Dong-Chan Park). It has been reported that the extracellular polysaccharides isolated from* Aureobasidium pullulans* are pullulan (poly-*α*-1,6-maltotriose-based exopolysaccharide, EPS) [20], aubasidan (*β*-1,3-D-glucan with *β*-1,6-branches to *α*-1,4 side chains) [21], and *β*-glucan (*β*-1,3/1,6-D-glucan) [22]. However, the physiological activity of polysaccharides produced by* Aureobasidium pullulans* has been mainly studied for  *β*-1,3/1,6-D-glucan [12]. Exopolymers purified from* Aureobasidium pullulans* SM-2001 (EAP; its solubility is about 10% in water), the test food material used in this study, containing 13%  *β*-1,3/1,6-glucan [23], are used in foods and have shown potent immunomodulatory activities in a mouse model [16]. In addition, EAP showed no adverse events in clinical studies designed to evaluate its effectiveness on bone metabolism improvement [24, 25] and on atopic dermatitis [26]. Therefore, *β*-glucan is considered safe for human ingestion.

Many reports have described the immunomodulatory activities of *β*-glucan against immune-related diseases [12–19], but only a few reports have described its effects against colds and flu. For this reason, a clinical study is now being performed to evaluate the efficacy and safety of *β*-glucan from* Aureobasidium pullulans* against colds and flu in patients.

In this study, we selected adult subjects who provided written, informed consent to participate, who satisfied our selection criteria, and who did not meet exclusion criteria. We randomly administered EAP or a placebo, where subjects took a daily dosage of 150 mg for 8 weeks. We evaluated immunological improvements of the test food by observing functional changes in the immune system before and after administering the test food materials in the EAP and placebo groups. We also observed the duration and total levels of cold and flu symptoms during the test period.

## 2. Materials and Methods

### 2.1. Test Materials

The EAP and placebo products used in this clinical research study were packaged inside boxes provided by Glucan Co., Ltd. (Busan, Korea) and were supplied and distributed by AriBio Co., Ltd. (Seongnam-si, Gyeonggi-do, Korea). Some EAP samples were deposited in the herbarium of Silla University (Busan, Korea; encoded as EAP2015Choi01). Sixty-five capsules were provided to each subject, taking into consideration an 8-week dosage (56 days, 56 capsules), a visitation window of 5 days (5 capsules), and storage products (4 capsules). The test food was manufactured as maroon-colored hard capsules, and each capsule of test food contained 150 mg of EAP and 100.0 mg of microcrystalline cellulose, whereas the placebo food contained 220.0 mg of microcrystalline cellulose. The test food was supplied to a testing agency in sealed white plastic bottles, each containing 65 capsules intended for 1 person.

The test foods used in this study were identical in appearance and description and would be difficult to distinguish with the naked eye. The experiment was conducted in double-blind format for the subjects and researchers by attaching identical labels to the test foods. The main ingredients, generic names, and lot number were not recorded on the test food labels to avoid exposing the differences between the test groups. Subjects took 1 EAP hard capsule once daily for 8 weeks on an empty stomach, with a sufficient amount of water.

### 2.2. Study Population

**Figure 1:**
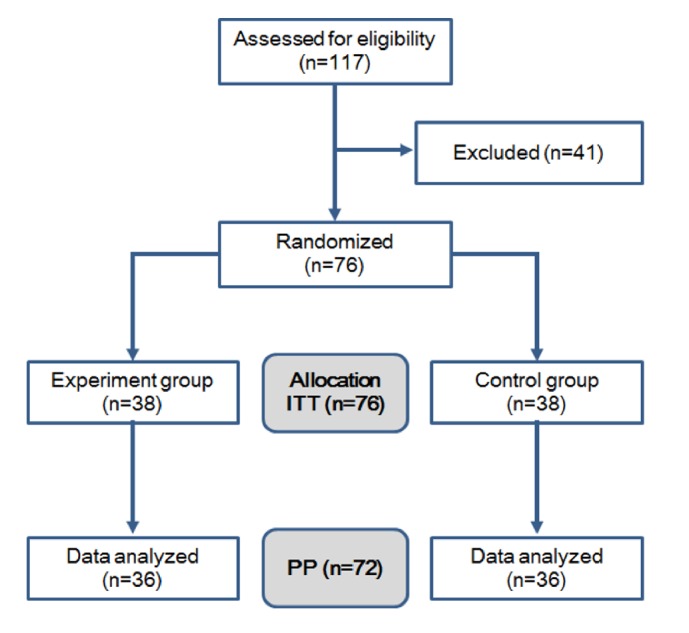
Schematic representation of this clinical study. ITT, intention to treat; PP, per protocol.

The subjects were healthy adults who did not have any disease that required proactive treatment. The number of subjects assigned to each group was 38 (76 overall). In consideration of a potential 20% dropout rate, the number of subjects in each group would decrease to 30, the minimum number of subjects for an effectiveness analysis (Figure 1).

#### 2.2.1. Inclusion Criteria

Subjects included in this study had to meet the following criteria: adults between 30 and 70 years of age; individuals with a white blood cell (WBC) count of 4–7 × 10^3^/*μ*L; individuals who could be monitored throughout the test period; individuals who listened to a thorough explanation of the study's purpose and contents and voluntarily signed an informed consent form to participate in the experiment.

#### 2.2.2. Exclusion Criteria

Subjects for whom the following criteria were applicable were excluded from the experiment: individuals with a body mass index (BMI) under 18 or over 35; individuals who exceeded the normal maximum alanine transaminase and aspartate transaminase levels by 2-fold; females who were pregnant or were breast-feeding; females of childbearing age who did not agree to use contraceptives via medically proven methods (e.g., condoms, lubricant, and femidom) during the test period; individuals with a fasting plasma dextrose concentration over 126 mg/dL; individuals with high blood pressure (systolic blood pressure of 160 mm Hg or diastolic blood pressure of 100 mm Hg); individuals continuously using medicine that could affect the effectiveness assessment (hyperlipidemia medicine, steroid medicines, hormone medicines, immunosuppressants, and antibiotics); individuals who require continuous treatment for psychiatric disorders such as anorexia, depression, and manic depression; individuals with systemic diseases such as immunity-related diseases, serious hepatic and renal insufficiencies, malignant tumors, pulmonary disease, collagenosis, multiple sclerosis, allergic skin conditions, and other autoimmune diseases; individuals with a medical history of drugs and clinically significant allergic reactions; individuals with a history of gastrointestinal disorders that could affect the absorption of the test foods or a history of gastrointestinal surgery (excluding a simple appendectomy or hernia operation); individuals who consumed medicine or herbal medicines within a month of participation in the experiment which could affect immunity; individuals who participated in a different human study or clinical test and took experimental products within 3 months of participation in this experiment (excluding human studies with cosmetics); and individuals whom the researchers otherwise determined might have difficulty completing the experiment.

#### 2.2.3. Withdrawal

Dosages of the experimental products were temporarily suspended at instances where it was appropriate to stop the experiment for the welfare of the subjects.

#### 2.2.4. Temporary Suspension for the Treatment of Adverse Events That May Have Occurred

In the following cases, administration of the test foods was temporarily suspended: (a) a temporary interruption on the occurrence of an adverse reaction or for the treatment of an adverse reaction; (b) when showing adverse events related to the safety of the test foods; and (c) in case of showing acute reactions (allergies and hypersensitivity) to the test foods. However, in the case of a temporary interruption for the occurrence of an adverse reaction or for the treatment of an adverse reaction, if there was no problem in its compliance, the study was continued.

#### 2.2.5. Dropout

Cases that were processed as dropouts included those in which test foods were allocated and the subject or a legal representative withdrew consent from participation in the experiment, cases where results for a final inspection could not be obtained, cases where subjects did not adhere to the test protocol resulting in a significant effect on the test results, cases where there was an interruption in contact with the subject (i.e., when the subject could not be traced), cases where the subject failed to make visits, subjects for whom a disease was discovered which went unnoticed during the screening inspection, cases where compliance with usage of the test food fell below 80%, as assessed with the usage log, cases in which, without the direction of the researcher, the subject used a medicine or product while they were taking the test food which could affect the research results or interpretation of data, cases in which the subject had an average weekly drinking quantity as ethanol of 490 g/week throughout the test period, or cases where the lead researcher determined that the research proceedings were inappropriate or that there could be a significant effect on the safety of the subjects or the experiment results.

Subjects stopped taking test foods and were processed as dropouts for the following reasons, in which it was not possible to continue the human study: cases in which a serious adverse event occurred with the subject, cases in which an adverse event made participation in the experiment impossible, cases in which the researcher determined that the usage and observation of the test foods would be impertinent, or cases in which clinical tests were not possible due to the death of a subject or emergence of a disease.

Subjects who took the test foods and later dropped out of the study, where a portion or the entire estimate value of the final assessment (visit 3) was omitted, were included in the ITT group's analysis data. Missing values were processed after a dropout occurred using missing value processing method for effective assessments. The results of all subjects who dropped out were excluded from assessment of the per protocol (PP) group (Figure 1 and Table 1).

#### 2.2.6. Drinking, Diet, and Exercise among the Subjects

During the experiment, subjects maintained regular levels of drinking, diet, and exercise which were similar to levels prior to participation in the experiment. To verify this, on the day of base evaluations, we examined the dietary contents for 3 contiguous days prior to the visitation day and the weekly exercise types and times prior to the visitation day. The number of alcoholic drinks and the amount of ethanol consumed during the week prior to the visitation day were also examined.

Subjects kept a daily record of whether they consumed the test foods and recorded 3 contiguous days of their diets, including Saturday or Sunday. Exercise, alcoholic drinks, and test food consumption were recorded in a chart provided by the testing agency, a comparison was made against these quantities prior to participation in the experiment, and observations were made as to whether there were any changes that could have significantyl affected the experiment results.

### 2.3. Randomization and Blinding

Subjects were assigned to the EAP group (the group taking EAP) and the placebo group (the group taking the placebo) based on a randomized list produced with a random sampling table generated using Microsoft Excel 2010 on Microsoft Windows (Figure 1).

The exclusion criteria used to select subjects during the screening visit (visit 1) were verified, and individuals who met selection criteria and had met no exclusion criteria were selected as subjects for this study. Food materials were allocated to the subjects during visit 2. The subjects selected for the experiment were issued a subject number in the order of visits made during visit 2 and were provided with the test foods, which were randomly allocated. The foods used in the human test were provided only to the participating subjects, based on the process described in the human test protocol (Table 1). The study protocol was approved by the institutional review board (IRB) of the Daegu Korean Medicine Hospital (Daegu, Korea; IRB No. DHUMC-D-15019). All procedures were performed in accordance with the ethics standards of the Declaration of Helsinki and Good Clinical Practice Guidelines.

### 2.4. Basic Information and Vital Signs of Subjects

#### 2.4.1. Visitation Schedule

The visitation dates allowed for the set base visitation days + 5 additional days. Those who were selected as test subjects received the test foods within 14 days from the base evaluation date (selection inspection date) and began taking the test foods by the next morning.

#### 2.4.2. Basic Information of Subjects

Demographic data including the initials, age, gender, birthdate, weight, and height of the subjects and basic information such as medical history, medicinal intake history, combined treatment, concomitant drugs, smoking, drinking, and exercise habits were recorded in detail. The contents during the last 3 years were recorded in the medical histories, and contents during the last 4 weeks were recorded in the medicinal intake history. The types of exercise and the exercise times were examined for 1 week prior to the time of the visitation day. Diet and exercise were compared during the base evaluation period and experimental period and observed to determine whether any important changes occurred which could affect the test results. The number and quantity of alcoholic drinks consumed during the week preceding the visitation day were examined, and that information was converted into the number of grams of ethanol consumed and recorded.

#### 2.4.3. Vital Signs and Obesity Levels

Blood pressure, body temperature, and pulse were measured and recorded as vital signs. Obesity levels were measured using a body fat analyzer (Inbody 3.0, Biospace, Korea), and the BMI (kg/m^2^), percent body fat (%), waist-hip ratio, and visceral fat area (cm^2^) were recorded.

### 2.5. Test Items

Hematological examination of WBCs, red blood cells, hemoglobin levels, hematocrits, and platelets was performed using an automated analyzer (Sysmex XP-300; Sysmex, Kobe, Japan). The levels of blood urea nitrogen, creatinine, uric acid, total bilirubin, albumin, total protein, alkaline phosphatase, glucose, alanine transaminase, aspartate transaminase, gamma-glutamyl transferase, triglyceride, total cholesterol, high-density lipoprotein cholesterol, and low-density lipoprotein cholesterol were measured using a clinical chemistry analyzer (Dimension Xpand Plus; SIEMENS, Munich, Germany). All urine samples were stored in the dark at 4°C and were analyzed within two hours of collection. The pH, specific gravity, and protein and glucose levels were measured using a semiautomated analyzer (URiSCAN Pro II; YD Diagnostics, Seoul, Korea). Pregnancy testing was performed using a kit detecting human chorionic gonadotropin. Serum concentration of cytokines was analyzed by using hMAGLXsa (6 PLEX) Multiplex ELISA kit (R&D Systems, Minneapolis, MN). Cytokine levels were determined using Luminex 200 (Luminex, Austin, TX, USA) and the data were reported as median fluorescent intensities.

### 2.6. Preliminary/Postsurvey Comparisons

If immune functions improve, the disease duration and symptom levels should decrease due to increased resistance to viral diseases such as colds and flu. Accordingly, we examined the duration and levels of cold and flu symptoms after consuming the test food products as a functional index of immunological improvements. For the survey following the administration of test foods, a form was provided in which subjects made a daily record of their health conditions, with a daily record of their test food dosages. Nine symptoms were monitored to inspect the health conditions of the subjects in the survey: fever, chills, runny nose, nasal congestion, coughing, sneezing, sore throat, headache, and gastrointestinal symptoms (nausea, vomiting, diarrhea, and abdominal pain). Subjects marked ○ for “symptoms exist” and × for “no symptoms.” To record the symptom levels, subjects used a 4-step scoring point system, where they marked (0) for no symptoms, (1) for minor symptoms, (2) for moderate symptoms, and (3) for serious symptoms [27] and marked whether they took early leave or were absent from work or school due to symptoms.  Table 2 shows a detailed explanation of the criteria used for reporting symptom levels. To assess the effectiveness of the experimental food products, subjects kept daily records in their dosage logs of cold and flu symptom durations and symptom levels during the testing period.

### 2.7. Compliance Evaluation

Compliance with ingesting test foods was evaluated based on the food-usage logs. During the final visit, the number of remaining foods collected was compared with the usage log records. In cases where there were differences between the recorded and actual amounts of food taken, compliance was verified when the differences were small.

### 2.8. Effectiveness Assessment Variables

Primary effectiveness assessment variables included the duration and total score of symptom levels for cold and influenza. Secondary effectiveness assessment variables included serum IL-1*β*, IL-6, IL-8, IL-10, TNF-*α*, and INF-*γ* levels, as well as WBC counts and differential blood counts.

### 2.9. Safety Assessment

#### 2.9.1. Groups Evaluated

The targets of evaluation included subjects who had taken (even once) foods used in the human study and who had been visited at least one time, from whom safety data in terms of adverse events, vital sign values, or changes in clinical examination values were procured.

#### 2.9.2. Evaluation Parameters

Subjects were evaluated in terms of predicted side effects, adverse events and adverse drug reactions, abnormal changes in clinical examination values, and vital signs.

### 2.10. Evaluation Methodology

A comparison was made of the side effects, adverse drug reactions, and the manifestation and frequency of adverse events, with a cause-and-effect relationship with the test foods predicted to occur in the EAP and placebo groups. Differences between the groups were analyzed by performing a chi-squared test and Fisher's exact test.

### 2.11. Statistical Analysis

Statistical analysis for assessing the effectiveness of test foods was based on the Statistical Guidelines for Clinical Trials [28], and the statistical package used was the SAS (version 9.4) on Microsoft Windows. The statistical significance level was set at 5%. In other words, there was statistical significance if the *p* value (significance probability) was below 0.05. Statistical analysis was performed by focusing on significance comparisons between the EAP and placebo groups. The methodologies described below were followed for detailed analysis.

### 2.12. Analysis Sets

Analysis sets obtained from the test subjects were composed largely of an ITT analysis group and a PP analysis group. The ITT group comprised the primary data set used for effectiveness assessments, and the PP group was additionally analyzed to assess the effectiveness. Subjects in the ITT group included those who had taken the test food materials and whose assessment variables were measured at least once during visit 1. Subjects in the PP group included those in the ITT group who completed the test according to the study protocol.

### 2.13. Statistical Analysis Items

#### 2.13.1. Baseline Observation Items Prior to Food Ingestion

Items for a baseline homogeneity test between the test groups are gender, age, weight, height, obesity, alcoholic drink consumption, exercise, and smoking.

#### 2.13.2. Pre/Post Food Ingestion Observation Items

Items for a pre/post food ingestion homogeneity test within the test groups are weight, height, alcoholic drink ingestion, and exercise. Items for an effectiveness test are duration and symptom levels of cold and flu symptoms; serum IL-1*β*, IL-6, IL-8, IL-10, TNF-*α*, and INF-*γ* levels; and WBC and differential blood counts. Other observation items are clinical laboratory test items.

#### 2.13.3. Homogeneity Test

A homogeneity test was carried out between the EAP and placebo groups on the baseline observation items prior to the ingestion of test foods. In this study, chi-square method was conducted for category variables or fisher's exact test was performed if over 25% of the cells had an expected frequency of less than 5. In the homogeneity test for continuous variables, independent *t*-test was conducted. In addition, analytical methods were employed using the above homogeneity tests to investigate whether weight, obesity, alcoholic drink ingestion, or exercise could have affected the test results, due to changes in lifestyle habits during the test period.

#### 2.13.4. Effect Test

It has been predicted that fewer subjects will contract viral diseases such as cold and flu if their immune functions are enhanced and that the duration and levels of symptoms will decrease if one of the diseases is contracted. The activation state of principal cells related to immunity can be determined by measuring changes in the serum contents of cytokine IL-1*β*, IL-6, IL-8, IL-10, TNF-*α*, and INF-*γ*. To assess the effectiveness of the test food materials, the significance of the mean difference (visits 1–3) was tested for each variable included in the effectiveness test. In other words, a statistical comparison was made between the average variation before and after food ingestions in the EAP and placebo groups. To this end, normality was tested for the evaluation data. The parametric method of a two-sample* t*-test was chosen if normality was satisfied, whereas the nonparametric method of a Wilcoxon's rank-sum test was chosen if normality was not satisfied. In addition, the above significance tests were performed on mean differences between the EAP and placebo groups between each visit after test food ingestions. To analyze the prevalence of cold symptoms, subjects recorded the duration and levels of symptoms when they contracted a cold or the flu prior to ingesting the test food. During visit 3, subjects recorded the duration and total symptom scores of cold and influenza symptoms during the test period. Accordingly, it was not meaningful to compare the mean difference between visits, considering that the rating scales used in visits 1 and 3 were different. In other words, we confirmed homogeneity between the test groups in visit 1 and evaluated the effectiveness by comparing the results between test groups in visit 3.

#### 2.13.5. Analysis of Missing Values

For the ITT group, an analysis is often conducted with the Last Observation Carried Forward (LOCF) method (a single-imputation method) for data missing in the final effectiveness assessment. Typically, the LOCF method is easy to use in clinical trials, despite the restricting assumption where missing data are treated as a constant. The LOCF method is commonly used because it provides conservative results. However, there are no commonly recommended methodologies for processing missing values [29]. In this study, the statistical analytic data collected was comprised of only the baseline assessment prior to the ingestion of test food materials and the final assessment after ingestion of test food materials. The missing values during the final assessment were not replaced by the base assessment values but were substituted by the average of each group and assessed.

## 3. Results

### 3.1. Subjects Participating in This Study

In this study, we screened 117 individuals who voluntarily agreed to participate in this human study, and 76 subjects who met the selection criteria and did not meet any exclusionary criteria were enrolled. Among the 76 subjects, 38 were assigned to the EAP group and 38 were assigned to the placebo group (Figure 1).

### 3.2. Violations of the Study Protocol

Four instances were verified in which subjects violated the study protocol while consuming the test foods. These 4 subjects were processed as dropouts.

### 3.3. Dropouts

Among the 76 subjects who were selected, 2 individuals from the EAP group dropped out due to violations in the visitation schedule and shortfalls in compliance, and 2 individuals from the placebo group dropped out due to withdrawal of informed consent and shortfalls in compliance. The remaining 72 subjects adhered to the test protocol and completed the test. The current states of dropouts are separated according to the test group (Figure 1).

### 3.4. Compliance Assessment

Compliance with ingesting test food materials was assessed in visit 3. The contents recorded in the daily record filled out by the subjects as well as the quantity of remaining products returned during the final visit were confirmed and assessed. Compliance was calculated using the following formula: compliance (%) = (number of food materials ingested/number of food materials that should have been ingested) × 100, where the number of food materials ingested = number of food materials provided − number of food materials returned.

Subjects whose compliance was below 80% were set as dropouts, and there were 2 dropouts in this study due to compliance shortfalls. The compliance of test food material ingestion was 93.82 ± 6.73% in the EAP group and 94.81 ± 6.09% in the placebo group. A significant difference in compliance between the test groups was not observed (Table 3).

### 3.5. Homogeneity Test

A homogeneity test was conducted between test groups for the following: measurement values prior to food material ingestion regarding demographic information of the subjects including age, gender, medical history, treatment history, medication history, and concomitant medication and measurement values before and after ingestion of food materials regarding items that could affect the test results based on changes in lifestyle habits during the test period including regular exercise, smoking, and drinking. The results of a preliminary investigation on the age, gender, medical history, treatment history, medication history, concomitant medication, exercise, smoking, and drinking among the subjects are summarized in Tables 3 and 4. No significant differences were found between the test groups regarding any of the items examined.

### 3.6. Analyzing the Results of Primary Effectiveness Assessment Variables in the ITT Group

**Figure 2:**
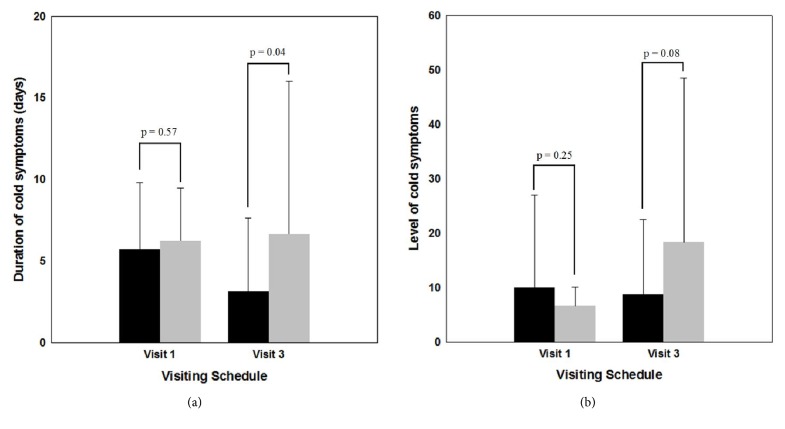
Foundational statistics and comparisons of cold symptom durations (days; (a)) and levels (b) between test groups: EAP group (black columns) and placebo group (gray columns).

The results of a *t*-test performed to evaluate significant differences between 2 groups before and after ingesting the test food materials verified that the duration of cold symptoms (*p* value = 0.5748) and cold symptom levels (*p* value = 0.2462) at visit 1 were not significant at a significance level of 5%. Statistical analysis also showed that no difference occurred between test groups prior to the ingestion of food materials. During visit 3, the duration of cold symptoms in the experiment group (3.1579 ± 4.4814 days) was lower than that of the placebo group (6.6842 ± 9.3581 days). The difference between the test groups was statistically significant (*p* value = 0.0410). The EAP group also had a lower score for cold symptom levels than the placebo group (8.7105 ± 13.7269 versus 18.3421 ± 30.1566, resp.). This difference was significant at a level of 10% (*p* value = 0.0790) but not 5% (Figure 2).

### 3.7. Analysis of Secondary Effectiveness Assessment Variables in the ITT Group

#### 3.7.1. Blood Test Results

Significant differences (visits 1–3) were verified with respect to the mean difference of each measured item between the visitation times to assess the effectiveness of the test food materials on the blood test results. The results were not significantly different between the EAP and placebo groups (Table 5) at a significance level of 5%. For WBC, basophil, and lymphocyte counts that did not follow normality, a nonparametric Wilcoxon's rank-sum test was conducted. The results were not significantly different between the EAP and placebo groups (Table 6) at a significance level of 5%.

#### 3.7.2. Cytokine Test Results

To assess the effectiveness of the test food materials, a *t*-test was conducted to evaluate significance differences in the mean cytokine concentrations between visits 1–3. The results were not significantly different between the EAP and placebo groups (Table 7) at a significance level of 5%. IL-10, IL-6, IL-8, and TNF-*α* values did not satisfy normality; a nonparametric statistics Wilcoxon's rank-sum test was conducted, revealing no significant differences in any of the items at a significance level of 5% (Table 8).

### 3.8. Safety Evaluation Results

#### 3.8.1. Adverse Events

Adverse events were observed in 3 individuals from the placebo group and in 1 individual from the EAP group. In the placebo group, nausea and pruritus were confirmed in subject E11, asthenopia and rash in subject E49, and a sore throat in subject E68. No other adverse events were observed aside from these. In the EAP group, headache was confirmed in subject E59, but no other adverse events were confirmed aside from this. All these adverse events subsided within a few days. In the case of subject E59, who ingested the test food materials, the headache was completely alleviated after 2 days, and the subject was judged as having no cause-and-effect relationship with ingesting the test food materials (data not shown).

#### 3.8.2. Abnormal Changes in Clinical Test Results

To study abnormal changes in clinical test results, we examined items in which blood biochemical results exceeded the normal range by 2-fold after the ingestion of test food materials. The results demonstrated changes in *γ*-GTP values in 1 individual from the placebo group, as well as changes in serum glutamic oxaloacetic transaminase (SGOT), serum glutamic pyruvic transaminase (SGPT), triglycerides (TG), and *γ*-GTP values in 4 individuals from the experiment group. In the placebo group, subject E62 demonstrated *γ*-GTP values during visit 3 (120 IU/L) which exceeded the normal range, similar to visit 1 (133 IU/L), but this was determined to be unrelated to the ingestion of test food materials because the *γ*-GTP values exhibited a decreasing trend. In the EAP group, the TG values of subject E26 increased from 217 mg/dL (visit 1) to 342 mg/dL (visit 3). Results prior to ingesting test food materials were also outside of the normal range, and this was determined to be unrelated to ingesting test food materials because no differences were confirmed between visits regarding abnormal changes in clinical test results. SGOT and SGPT values in subjects E41 and E61 exceeded the normal range by 2-fold during visit 3 but not during visit 1. However, based on a training lecture titled "Assessing the Abnormal Liver Function Test" from the 2015 Korean Society for Health Promotion and Disease Prevention's Academy of Family Medicine, if the SGOT/SGPT ratio falls under 1 while the SGOT or SGPT exceeds normal upper limit by 3-fold, the finding is not considered to represent an abnormal phenomenon. However, the *γ*-GTP value was found to be abnormally high during visit 3 for subject E57, but examination of the results showed that the abnormal values were not related to the ingestion of test food materials. Instead, the results were due to excessive drinking, where the subject consumed alcoholic drinks frequently (5 days/week) and a high quantity of ethanol (average = g/week) (data not shown).

#### 3.8.3. Overall Safety Level

With the occurrence of adverse events and abnormal changes in clinical test results, no items were found to correlate with the use of test food materials or were suspected to have a correlation. Thus, the test foods were found to be safe in the context of this study.

## 4. Discussion

### 4.1. Effects of EAP against Colds and Flu

The various biological activities of *β*-glucan were verified through* in vitro* testing, animal testing, and clinical trials [12]. Recently, the effectiveness of *β*-glucan on respiratory diseases has been studied in rodent [30] and human [31–33] because their prevalence has increased rapidly. EAP, exopolymers purified from* Aureobasidium pullulans* SM-2001, containing 13% *β*-1,3/1,6-glucan [20], are used in test foods and have shown potent immunomodulatory activities in a mouse model [16]. However, this study is the first to evaluate the use of EAP against respiratory diseases.

To assess the ability of EAP to improve human immunological responses and to alleviate cold or flu symptoms, 117 adults aged 30–70 years with a white blood cell count of 4–7 x 10^3^/*μ*l were screened. 76 individuals were enrolled as subjects who satisfied the selection criteria and did not fall under any exclusion criteria (data not shown). In an aspect of subject's respiratory morbidity, none of the subjects had a respiratory disease at the time of enrollment. Within 4 weeks prior to the screening date of the subjects, there were 2 subjects with light respiratory disease. After confirming no symptoms at the enrolment, the 2 subjects were proceeded to obtain informed consent.

The 76 subjects (administrated with the test foods for a period of 8 weeks) were randomly assigned to either EAP or a placebo group. Blood tests and surveys of cold or flu symptoms were conducted before and after ingesting test food materials, and the duration and symptom level of colds or flu, as well as the results of the blood test and cytokine analysis, were compared. The EAP and placebo groups were composed of individuals that exhibited homogeneity and showed no statistically significant differences in their compliance of ingesting test foods or in gender, age, compliance, regular exercise, smoking, drinking, medical history, medicinal history, or concomitant drug administration.

Clinical data have shown that immunity-promoting functions can prevent acute respiratory infections from cold or influenza viruses and reduce the frequency of contracting colds or flu, symptom levels, and symptom durations based on effectiveness criteria [34]. In this study, statistical analysis of the duration and level of cold symptoms, a primary variable for assessing effectiveness, showed that there were no differences in assessment values between the EAP and placebo groups before ingesting test food materials.

However, the duration of cold and flu symptoms after ingesting test food materials was significantly lower in the EAP group than in the placebo group at a 5% significance level. The EAP group also showed lower levels of cold and flu symptoms, which were statistically different at the 10% (but not 5%) significance level than placebo group.

It is predicted that individuals whose immune functions are enhanced will contract fewer viral diseases such as colds and flu, with reduced durations and levels of symptoms. The activation state of primary cells related to immunity can be determined by measuring the serum levels of the cytokines IL-1*β*, IL-6, IL-8, IL-10, TNF-*α*, and INF-*γ* [1]. According to the “Guideline for Safety and Efficacy Assessments of Functional Food on Immune Function Improvement” from the Ministry of Food and Drug Safety of Korea, changes in cytokine levels, WBC counts, T cell counts, and NK cell activity in the peripheral blood should be measured when determining the functionality of immunity reinforcement in humans [35].

In this study, secondary variables including serum cytokines (IL-1*β*, IL-6, IL-8, IL-10, TNF-*α*, and INF-*γ*) and WBCs (neutrophil, eosinophil, basophil, lymphocyte, and monocyte) for assessing effectiveness did not exhibit statistically significant differences before and after ingestion of test food materials at a significance level of 5%. These results are consistent with those of Choi et al. (2009) [14], where the daily intake level of EAP (Polycan^TM^) or placebo food materials was set at 400 mg, and changes in serum cytokines were studied (with the intention of observing effectiveness in immunity improvements of EAP), but no statistically significant changes were found.

Although EAP has shown potent immunomodulatory activities in a mouse model [16], no immunity improvement of EAP on the human was observed in this study. The cytokine is a factor that needs to change suddenly according to the individual health condition and then return to the normal range [1]. On the normal range of blood cytokine concentration, its individual variation is very large. Thus, a large number of subjects are required to measure the normal range of cytokine concentrations [36]. Indeed, in a study of normal Korean cytokine levels, 110 subjects were studied [37], and in a study of normal American cytokine levels, 144 subjects were examined [38] to estimate the normal range of blood cytokine concentration. Serval studies have shown that no changes in blood cytokine levels have been observed following ingestion of multivitamin and mineral supplement [39], probiotic supplement [40], fish oil [41], and* Cordyceps militaris* [42], which are known to help improve immunity. This may be because the cytokine level increased during the test period and then returned to normal, or the number of subjects was small, indicating that the individual deviation range was not statistically significant. In this study, the total number of subjects was 72: 36 in the test group and 36 in the control group, respectively. Therefore, the results of this study may not statistically overcome the individual variation range of the subject's cytokine level due to the small number of subjects. Further research is needed on extensive clinical studies with clear statistical numberings to confirm the effects of EAP on plasma cytokines.

No significant adverse events occurred during the period of test food material intake, and adverse events (excluding colds) were observed in 3 individuals from the placebo group (nausea, pruritus, asthenopia, rash, and sore throat) and in 1 individual from the experiment group (headache). All these adverse events subsided within just a few days and were determined to have no cause-and-effect relationship with the ingestion of test food materials. Changes in blood test values were also observed in the EAP group with an increase in SGPT, TG, and *γ*-GTP values, but this was difficult to determine as being clinically abnormal. In addition, in an aspect of the respiratory morbidity, the subjects who suffered from cold during the test period appeared and then fully cured after the treatment, and no respiratory morbidity was shown in all subjects. Therefore, EAP was verified to be harmless to the human body.

### 4.2. Conclusion

Collectively, our data revealed that EAP was associated with a statistically significant decrease in the duration of cold and flu symptoms, a primary variable in assessing effectiveness. Although cold symptom levels were not different at a significance level of 5%, the cold and flu symptom levels of the EAP group were less severe compared to the placebo group. No statistically significant changes were observed in the serum cytokine concentrations, WBC, and differential blood counts (secondary variables for assessing effectiveness). Therefore, our data showed that EAP is a useful medicine and functional food material for preventing and treating colds and flu.

## Figures and Tables

**Table 1:** Experiment schedule in this clinical study.

Visiting day Verification/assessment items	Visit 1	Visit 2	Visit 3
	Screening base evaluation	Product provision	Test completion, final assessment (8 weeks)
	–14 days to 0 days	0 days	56 days + 5 days
Informed consent form	○		
Demographic study	○		
Medical history/drug administration history	○		
Vital sign test	○	○	○
Preliminary/postsurvey	○		○
Body composition test	○		○
Clinical laboratory test	○		○
Urinalysis	○		○
Cytokine analysis	○		○
Pregnancy test	○		
Inclusion/exclusion criteria review	○		
Assignment of test foods		○	
Adverse event monitoring	○	○	○
Concomitant drugs check	○	○	○
Compliance examination			○

**Table 2:** Symptom level of illness, such as cold and flu.

Symptom level	Score	Symptom levels reported by the patients
No symptom	0	I did not perceive relevant symptoms
Mild	1	I perceived some symptoms, but they did not interfere with my daily life
Moderate	2	I perceived symptoms and felt discomfort in my daily life. I was able to take general pharmaceuticals without causing an issue in my daily life
Severe	3	My daily life was very uncomfortable due to symptoms, and proactive treatment was needed. In the case of severe discomfort, I may stop working or studying or be admitted to the hospital

**Table 3:** Foundational statistics and homogeneity testing for compliance with ingesting test foods, subject age, number of days consuming alcoholic drinks, and quantity of alcoholic drinks.

Variable			EAP group		Placebo group		*t*-value	*p* value
			Mean	Std. dev.	Mean	Std. dev.		
Compliance			93.82	6.73	94.81	6.09	0.66	0.5140

Age			46.42	10.09	48.86	9.89	1.04	0.3026

Drinking	Visit 1	No. of days drinking	1.78	1.06	2.00	1.00	0.64	0.5285
		Amount of drinks consumed (g alcohol)	68.36	54.28	70.41	84.55	0.09	0.9321
	Visit 3	Total no. of days drinking	9.74	5.72	10.05	8.38	0.14	0.8928
		Amount of drinks consumed (g alcohol)	415.50	350.80	407.70	402.60	-0.07	0.9484

**Table 4:** Foundational statistics and homogeneity testing of subject gender, drinking, exercise, smoking, medical history, and medications.

Variable	Group		Male		Female		Chi-squared value	*p* value
			Frequency	%	Frequency	%		
Gender		EAP group	7	19.44	29	80.56	2.4923	0.1144
		Placebo group	13	36.11	23	63.89		

Drinking	Drinking (visit 1)	EAP group	18	50.00	18	50.00	0.0556	0.8136
		Placebo group	17	47.22	19	52.78		
	Drinking (visit 3)	EAP group	19	52.78	17	47.22	0.6353	0.6353
		Placebo group	21	58.33	15	41.67		

Variable	Group		Yes		No		Chi-squared value	*p* value
			Frequency	%	Frequency	%		

Exercise	Visit 1	EAP group	28	77.78	8	22.22	0.0000	1.0000
		Placebo group	28	77.78	8	22.22		
	Visit 3	EAP group	27	75.00	9	25.00	0.0000	1.0000
		Placebo group	27	75.00	9	25.00		

Smoking	EAP group		1	2.63	37	97.37	1.0588	0.3035
	Placebo group		3	8.33	33	91.67		

Medical history	EAP group		2	5.56	34	94.44	2.0571	0.1515
	Placebo group		0	0.00	36	100.00		

Medications	EAP group		1	2.78	35	97.22	1.9343	0.1643
	Placebo group		4	11.11	32	88.89		

Concomitant drug administration	EAP group		3	8.33	33	91.67	0.0000	1.0000
	Placebo group		3	8.33	33	91.67		

**Table 5:** Foundational statistics and comparisons of blood test results between test groups.

Variable	Group	Mean	Std. dev.	*T*-value	*p* value
White blood cell	EAP group	–0.41	1.07	0.31	0.6225
	Placebo group	–0.32	1.25		

Neutrophil	EAP group	–0.079	8.07	–0.39	0.3489
	Placebo group	–0.81	8.29		

Eosinophil	EAP group	–0.04	1.19	–0.07	0.4717
	Placebo group	–0.06	0.93		

Basophil	EAP group	–0.05	0.27	–0.82	0.2086
	Placebo group	–0.10	0.26		

Lymphocyte	EAP group	–0.38	6.90	0.68	0.7491
	Placebo group	0.70	7.10		

Monocyte	EAP group	0.55	2.25	–0.53	0.2973
	Placebo group	0.2676	2.40		

Missing values were replaced with averages and used in the ITT group data analysis.

**Table 6:** Comparison (nonparametric statistical analysis) of the median of the subjects' blood test results.

Variable	Group	Expected value under H0	Std. dev. under H0	Mean score	*Z*-value	*p* value
White blood cell	EAP group	1,463	96.21	37.00	–0.5872	0.2785
	Placebo group	1,463	96.21	40.00		

Basophil	EAP group	1,463	95.83	40.13	0.6418	0.2605
	Placebo group	1,463	95.83	36.87		

Lymphocyte	EAP group	1,463	96.26	37.41	–0.4259	0.3351
	Placebo group	1,463	96.26	39.60		

Wilcoxon's rank-sum test was used to analyze variables that did not follow normality.

**Table 7:** Foundational statistics and comparison between test groups in terms of serum cytokine levels.

Variable	Group	Mean	Std. dev.	*T*-value	*p* value
IFN-*γ*	EAP group	–1.12	6.57	0.36	0.6398
	Placebo group	–0.51	8.07		

IL-1*β*	EAP group	0.52	2.28	–0.26	0.3987
	Placebo group	0.40	1.96		

IL-10	EAP group	0.31	1.29	–0.81	0.2107
	Placebo group	–0.24	4.01		

IL-6	EAP group	0.21	0.78	–0.93	0.1782
	Placebo group	0.01	1.07		

IL-8	EAP group	–10.53	47.64	1.18	0.8772
	Placebo group	–1.36	5.00		

TNF-*α*	EAP group	0.96	1.41	–0.69	0.2464
	Placebo group	0.74	1.31		

Missing values were replaced with averages and used in the ITT group data analysis.

**Table 8:** Comparison (nonparametric statistical analysis) of serum cytokine levels.

Variable	Group	Expected value under H0	Std. dev. under H0	Mean score	*Z*-value	*p* value
IL-10	EAP group	1,463	96.26	40.43	0.7584	0.2241
	Placebo group	1,463	96.26	36.57		

IL-6	EAP group	1,463	96.24	39.86	0.5299	0.2981
	Placebo group	1,463	96.24	37.14		

IL-8	EAP group	1,463	96.26	36.58	-0.7532	0.2257
	Placebo group	1,463	96.26	40.42		

TNF-*α*	EAP group	1,463	96.20	39.64	0.447	0.3275
	Placebo group	1,463	96.20	37.36		

A Wilcoxon's rank-sum test was used for variables that do not follow normality.

## Data Availability

The data used to support the findings of this study are available from the corresponding author upon request.
